# Mechanisms of chemotherapeutic resistance and the application of targeted nanoparticles for enhanced chemotherapy in colorectal cancer

**DOI:** 10.1186/s12951-022-01586-4

**Published:** 2022-08-11

**Authors:** Yu Guo, Min Wang, Yongbo Zou, Longhai Jin, Zeyun Zhao, Qi Liu, Shuang Wang, Jiannan Li

**Affiliations:** 1 Department of the General Surgery, Jilin University Second Hospital, Changchun, 130000 China; 2 Department of Radiology, Jilin University Second Hospital, Changchun, 130000 China; 3 Department of the Dermatology, Jilin University Second Hospital, Changchun, 130000 China

**Keywords:** Colorectal cancer, Targeted nanoparticles, Chemotherapeutic resistance

## Abstract

Colorectal cancer is considered one of the major malignancies that threaten the lives and health of people around the world. Patients with CRC are prone to post-operative local recurrence or metastasis, and some patients are advanced at the time of diagnosis and have no chance for complete surgical resection. These factors make chemotherapy an indispensable and important tool in treating CRC. However, the complex composition of the tumor microenvironment and the interaction of cellular and interstitial components constitute a tumor tissue with high cell density, dense extracellular matrix, and high osmotic pressure, inevitably preventing chemotherapeutic drugs from entering and acting on tumor cells. As a result, a novel drug carrier system with targeted nanoparticles has been applied to tumor therapy. It can change the physicochemical properties of drugs, facilitate the crossing of drug molecules through physiological and pathological tissue barriers, and increase the local concentration of nanomedicines at lesion sites. In addition to improving drug efficacy, targeted nanoparticles also reduce side effects, enabling safer and more effective disease diagnosis and treatment and improving bioavailability. In this review, we discuss the mechanisms by which infiltrating cells and other stromal components of the tumor microenvironment comprise barriers to chemotherapy in colorectal cancer. The research and application of targeted nanoparticles in CRC treatment are also classified.

## Introduction

Colorectal cancer (CRC) is considered one of the major malignancies threatening the lives and health of people around the world. In 2020, more than 1.93 million additional CRC cases and 930,000 malignancy-related deaths worldwide were reported, accounting for 9.7% of all new cancer detections [1]. CRC is caused by factors related to lifestyle, diet, and genetic mutations [2]. Obesity, smoking, red meat, and heavy alcohol consumption are considered high-risk factors for CRC, while dietary fiber and aspirin are protective factors [3, 4]. Surgery is the preferred treatment for patients with early-stage CRC, in which healing is achieved by removing the tumor site and a portion of the healthy intestine [5]. Post-operative adjuvant chemotherapy in early-stage patients can effectively eliminate residual micro-metastases, eradicate local implants during surgery, and reduce the probability of recurrence. For patients with advanced disease or unresectable tumors, radiotherapy and chemotherapy are highly effective adjuvant therapies to reduce the tumor load, thus achieving clinical resection and prolonging the overall survival of patients [6, 7]. 5-Fluorouracil (5-Fu) and oxaliplatin are the first-line agents for CRC chemotherapy, and 5-Fu-based chemotherapy regimens are widely used in CRC patients. The chemotherapy agents have poor selectivity and damage massive normal cells while killing tumor cells. These injuries can cause damage to normal tissues and organs, such as inhibiting the hematopoietic function of bone marrow, damaging liver cells, and inducing gastrointestinal reactions such as nausea and vomiting [8, 9]. Since it is difficult for the drugs in conventional formulations to effectively enter the tumor tissues, higher doses are required to achieve therapeutic effects, and the drug toxicity caused by the high dose should not be neglected [10]. The specific delivery of drug carriers can be exploited to target chemotherapeutic drugs to tumor sites, thereby enhancing drug treatment effects and reducing drug-related toxicity.

Tumor tissue includes tumor cells, tumor stem cells, and the microenvironment in which the cells reside. Tumor formation is frequently accompanied by the formation of a tumor bed and deep changes in the surrounding connective tissue and stroma, creating a microenvironment suitable for tumor cell survival [11]. The tumor microenvironment (TME) consists of a complex network of multiple stromal cells and extracellular components. Stromal cells primarily include mesenchymal stem cells (MSCs), cancer-associated fibroblasts (CAFs), endothelial cells, immune cells, and adipocytes. The extracellular matrix (ECM) is a non-cellular three-dimensional macromolecular network composed of collagen, proteoglycans (PGs), glycosaminoglycans (GAG), elastin, fibronectin (FN), laminin (LN) and several other glycoproteins, providing structural and mechanical support and protection to cells [12]. ECM regulates cell proliferation and survival, cell differentiation, cell migration and invasion, and tissue morphogenesis. The rigid ECM creates a physical barrier to the entry of chemotherapeutic agents and inhibits their diffusion into cancer cells [13]. Alterations in the tumor stroma promote cancer progression and metastasis, leading to disease recurrence and resistance to therapy. In addition, other extracellular components such as various enzymes and growth factors also participate in tumor development.

Nanoparticles (NPs) based on nanotechnology have attracted the attention of researchers. It penetrates the barriers of high cell density, dense extracellular matrix, and high osmotic pressure of tumor tissues, enabling efficient targeting of chemotherapeutic drugs to tumor tissues [14]. As a drug delivery system (DDS), NPs can improve the pharmacokinetics of drug molecules by altering their physicochemical properties, thus addressing physiological and pathological problems. At the same time, active and passive targeting can be realized to increase the local concentration of nanomedicines in the lesion. It improves drug efficacy and reduces side effects, allowing safer and more effective disease diagnosis and treatment [15]. Targeted DDS employing NP carriers generally refers to administration via vascular injection. By enhancing passive targeting with permeation retention (EPR) effects and active targeting with specific ligand–receptor binding, the proportion of nanomedicines reaching the target site can be increased. The NPs can efficiently deliver drugs to the cell interior. NPs smaller than 10 nm will be quickly cleared into the urine through the tiny pores on the kidney, while those larger than 10 nm will ensure an effective long circulation time in vivo. Therefore, spherical NPs with less deformable shape and proper size are crucial for the successful delivery and penetration of anticancer drugs, which is directly related to the endocytosis-based cellular internalization process [16, 17]. In addition, a significant proportion of natural and synthetic NPs possess favorable biocompatibility and utilization, making them one of the necessary conditions for effective DDS. Based on these advantages, NPs are believed to penetrate the chemotherapeutic barrier of the CRC microenvironment and become a compelling DDS.

In this review, the cause of chemotherapeutic drug barrier formation in TME is analyzed based on changes in its stromal cells, non-cellular components, and various factors. Furthermore, current research on targeted NPs as a chemotherapeutic DDS for CRC is discussed, as well as the current challenges and prospects.

## TME and chemotherapy obstacles

**Fig. 1:**
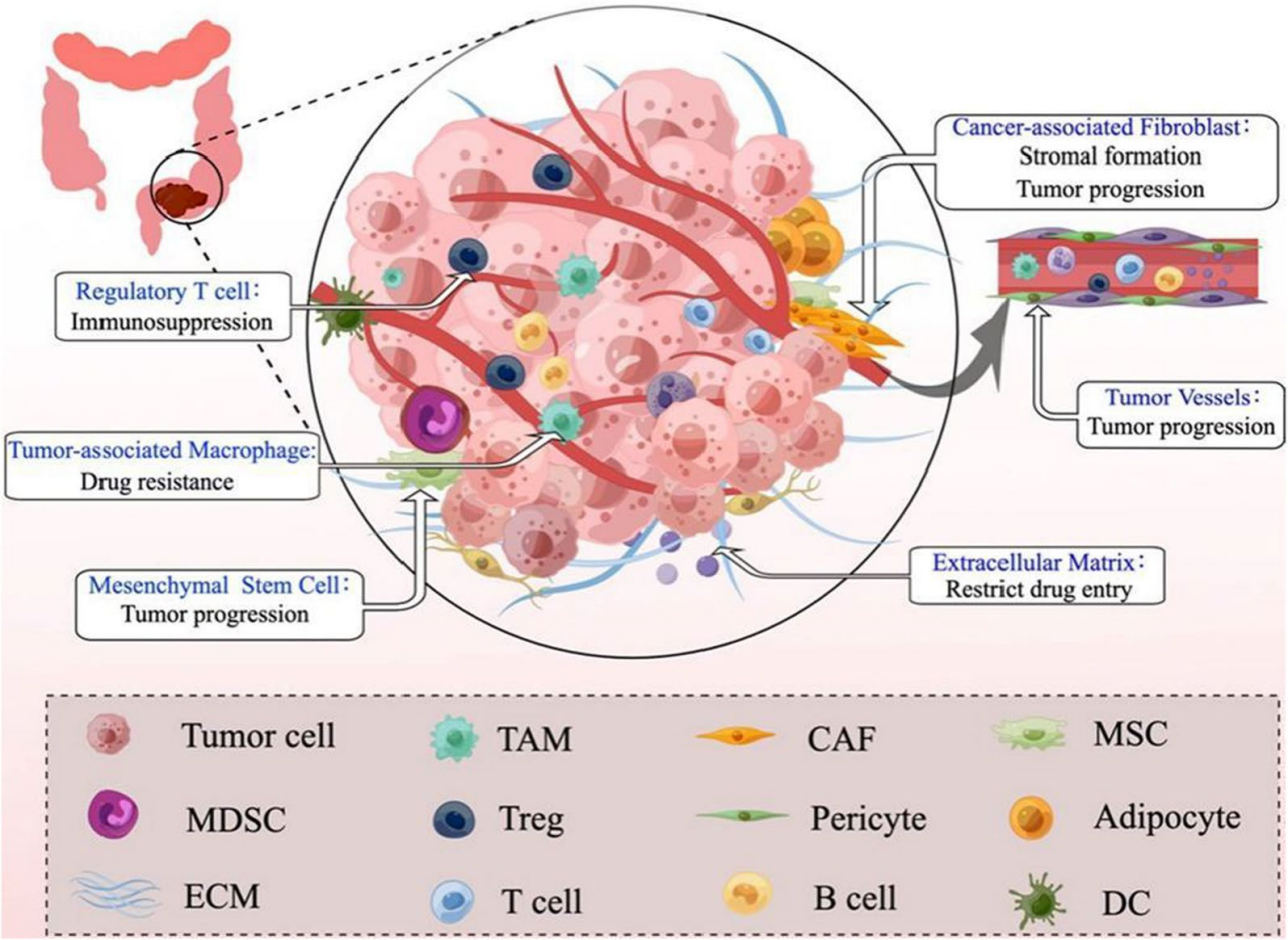
The complex microenvironment of tumors and its role in tumor progression

TME, including both stromal cells and extracellular components, is the internal environment in which tumor cells emerge and reside. The stromal cells and extracellular components interact with each other and co-evolve to promote tumorigenesis and progression (Fig. 1). The typical features of TME are high fibrosis and suppressive immune cell infiltration, preventing the entry of conventional chemotherapeutic agents into tumor cells and leading to the development of drug resistance [18]. Tumor cells promote the aggregation of fibroblasts, migration of immune cells, remodeling of the stroma, and formation of vascular networks through the secretion of cytokines. These activities contribute to the formation of TMEs that sustain the survival of tumor cells, and TME in turn plays an integral role in tumorigenesis and progression [19, 20]. In all, the chemotherapy obstacles were closely related to TME stromal cells, ECM, and the special biochemical environment of TME.

### TME stromal cells and chemotherapy obstacles

Tumor cells are a major component of tumor tissue, and they frequently develop drug resistance during chemotherapy. Tumor resistance is the leading challenge that limits the efficacy of current cancer chemotherapy drugs [21]. There are two forms of tumor drug resistance: intrinsic resistance and acquired resistance [22]. Many tumor patients have remarkable treatment efficacy in the early stage of chemotherapy, but with the prolongation of treatment, the resistance of tumor cells to chemotherapeutic agents increases, which eventually leads to treatment failure. The development of acquired drug resistance in tumor cells may be associated with multiple mechanisms: (1) Increased drug excretion. ATP-binding cassette (ABC) transporter protein family is a widespread transmembrane protein found on the surface of multiple biological cells, whose primary function is to transport various substances across the cell membrane [23]. This efflux mechanism is instrumental in preventing the excessive accumulation of intracellular toxins. Multidrug resistance protein 1 (MDR1), which produces P-glycoprotein (Pgp), has elevated gene expression in response to tissue carcinogenesis in the colon, liver, and kidney [24]. Pgp actively pumps drugs out of cells, keeping drug concentration relatively low in tumor cells [25]. Wang et al. reversed the resistance of tumor cells to 5-Fu by targeting miR-26b to downregulate Pgp expression in HT-29 and LOVO tumor cells [26]. (2) Changes in drug target. The treatment efficacy of certain agents can be influenced by changes of the molecular target and target alterations. Mutations or altered expression levels of target genes may result in drug resistance. For example, sorafenib, a multi-kinase inhibitor that acts on epidermal growth factor receptor (EGFR) family members and downstream signaling partners, tends to achieve better efficacy when applied to BRAF wild-type CRC patients [27]. However, the BRAF V600E mutation is present in approximately 10% of CRC tumors and exhibits unsuppressed cell proliferation, reduced apoptosis, and resistance to BRAF-targeted inhibitors [28]. CRC patients with KRAS mutant phenotypes tend to exhibit resistance to anti-EGFR monoclonal-based therapy [29]. (3) DNA damage repair (DDR). For chemotherapeutic drugs that damage DNA directly or indirectly, the DDR mechanism could reverse the drug-induced damage [30]. Chemotherapeutic drugs of platinum are cytotoxic which can damage DNA structure and act primarily during the DNA replication phase of cell division. Cisplatin enters the tumor cells and forms cisplatin-DNA adducts with DNA, destroying the normal structure of DNA [31]. However, tumor cells initiate a variety of different DNA damage repair pathways (including direct repair, base excision repair, nucleotide excision repair, DNA mismatch repair and double-strand break damage repair) to rapidly repair DNA damage caused by platinum, allowing cells to re-enter mitosis and leading to resistance to platinum drugs [32]. DDR inhibitors may benefit CRC patients by modulating the sensitivity of tumor cells to oxaliplatin or cisplatin [33]. (4) Heterogeneity includes both inter-tumor and intra-tumor heterogeneity, specified as differences between patients for the same type of tumor and differences between various tumor cells within the same tumor. It mainly includes differences in cell morphology, gene expression, proliferation, and metastatic potential. Heterogeneity explains the varying response of patients to treatment [34]. Sensitive cells are killed when chemotherapy drugs act on tumor cells, and yet subpopulations of tumor cells that are resistant to the drugs begin to proliferate, eventually leading to drug resistance [35]. The other factors such as the high mutation, fast division, and rapid metastatic ability of tumor cells also contribute to drug resistance in tumor cells.

MSCs are somatic stem cells with multiple differentiation potentials, which can be recruited by various chemokines secreted from CRC tumor cells, migrate to the tumor mesenchyme, and participate in the composition of TME. The homing process of MSC to tumor tissue mainly depends on the activation of multiple relevant receptors on MSC by cytokines and chemokines released from tumor tissue. The main chemokines are CXC subfamily ligand 12 (CXCL12), CXCL16, CCL19, TGF-β1, epidermal growth factor, and platelet-derived growth factor [36–38]. Fontanella et al. [39] co-cultured bone marrow-derived MSCs with a hepatoma cell line (SNU-398). The experimental results demonstrated that MSCs could induce tumor migration through AKT and ERK signaling pathways. When CXCR4 inhibitors were applied to downregulate the expression, the ability of MSCs to target hepatocellular carcinoma cells for migration was reduced. MSCs in tumor tissue have a critical impact on CRC development [36], angiogenesis, promotion of epithelial–mesenchymal transition (EMT) [37], chemotherapy resistance, and stemness maintenance [40]. The co-culture of MSCs with CRC cells was found to enhance the sphere-forming CRC cell ability in vitro and to promote CRC proliferation and metastasis. MSCs promote tumor development by secreting cytokines and growth factors such as interleukin-6 (IL-6) and TGF-β and vascular endothelial growth factor (VEGF) [37, 41]. The IL-6 secreted by MSCs could activate multiple signaling pathways, including JAK2/STAT3 [42], ERK/MAPK [43], and PI3K/AKT [44]. The activation enhances the recruitment of MSCs to tumor cells and angiogenesis. Among them, the activation of the STAT3 signaling pathway by IL-6 increases the number of CRC initiating cells, which can induce non-tumor stem cells to express stem cell markers and increase tumorigenic capacity in vivo [42]. Lin et al. [41] observed that human-derived MSCs isolated from CRC tissues significantly enhanced the migration, invasive activity, and tumorigenic ability of HCT116 cells in mice. Significant co-expression of IL-6, Notch-1, and CD44 was also detected in the tumor tissues. These results indicate the crucial effect of MSCs in promoting tumorigenesis, metastasis, and maintenance of stemness through the secretion of cytokines and growth factors. Furthermore, at least 20% of CAFs were found to be differentiated from MSCs. CAFs specifically express α-smooth muscle actin (αSMA) and platelet-derived growth factor receptor α (PDGFRα), which promote tumor metastasis and drug resistance, as well as forming a dense fibrous interstitium that encapsulates tumor tissue [45].

CAFs are the main cells involved in the formation of tumor stroma. They provide mechanical support to tumor cells and have a valuable effect on tumor cell survival, proliferation, metastasis, and chemoresistance. CAFs secrete various soluble ligands such as CXCL12, CCL7, and TGF-β, which may interact with tumor cells and promote tumor progression. CAFs are also involved in tumor recurrence, metastasis, and drug resistance through exosome secretion. Ren et al. [46] demonstrated that CAFs could secrete H19-containing exosomes to activate the Wnt/β-catenin signaling pathway in CRC cells, exhibiting stemness maintenance capacity and resistance to oxaliplatin. By secreting multiple cytokines and chemokines, CAFs facilitate immune escape (e.g., IL-6, IL-1, and FGF), recruit immunosuppressive cell infiltration [47], and suppress the ability of cytotoxic lymphocytes to kill tumor cells [48]. In addition, CAFs can further activate the Wnt signaling pathway and promote chemotherapy resistance through tumor stemness regulation. It was observed that Wnt signaling activity near CAFs was higher in CRC, and the activation of the Wnt signaling pathway could further promote the expression of downstream target genes c-Myc, CCND1, Sox4, and Lgr5. These genes were involved in tumor stemness regulation and enhanced the self-renewal and regulation of colorectal cancer stem cells [49, 50]. A variety of matrix metalloproteinases (MMPs) can be secreted by CAFs. The induction of MMP activity contributes to the disassembly of intercellular junctions and the degradation and remodeling of the ECM. In this way, the distant colonization and growth of tumor cells are facilitated, and the physical limitations of cell motility are broken, allowing cells to participate in tumor invasion [19, 51]. In addition to cytokines and chemokines, CAFs secrete abundant ECM stromal components that contribute to the formation of highly fibrotic tumor mesenchyme. The massive deposition of collagen, hyaluronic acid (HA), and fibrin adhesive proteins form highly fibrotic tumor mesenchyme, which acts as a physical barrier to the migration of cytotoxic T cells and the entry of chemotherapeutic agents [52].

Immunosuppressive cells recruited into TME are also involved in the development of immune tolerance. It has been reported that Regulatory T cells (Tregs)-mediated immunosuppression has a critical effect on autoimmunity, allergy, inflammation, and tumorigenesis. Tregs could secrete multiple suppressive cytokines, including TGF-β, IL-35, and IL-10 [53, 54]. TGF-β is a critical immunomodulatory cytokine closely associated with the induction, development, and maintenance of Tregs [55]. Salem et al. [56] proved that Treg-specific glycoprotein-A repetitions predominant (GARP) could bind and activate latent TGF-β involved in the formation of immunosuppression. The deletion of GARP on Tregs greatly enhanced the anti-tumorigenic capacity of CRC. IL-35 secreted by Tregs is involved in suppressing the inflammatory response of immune cells. Studies in mouse models have revealed that deletion of IL-35 reduces the ability of cells to suppress inflammation [57]. IL-10 suppresses the activation, migration, and adhesion of inflammatory cells, inhibiting inflammatory and cellular immune responses and restraining the production of pro-inflammatory factors by monocytes and macrophages [58]. Ning et al. [59] revealed a significant increase in miR-208b levels in the serum of oxaliplatin-resistant CRC patients. The in vitro experiments indicated that CRC cells could secrete miR-208b through exosomes to promote Tregs expansion by targeting programmed cell death factor 4 (PDCD4). This result might be related to the reduced chemosensitivity of CRC to oxaliplatin. γδT cells have always been the pivotal immunosuppressive cells hiding in CRC. CD39^+^ γδTregs are the predominant Treg subtype in human CRC. They act through adenosine-mediated pathways and have stronger immunosuppressive activity than CD4^+^ or CD8^+^ Tregs [60]. The Myeloid-derived suppressor cells (MDSC) can be attracted by the secretion of cytokines such as IL17A and GM-CSF, thus establishing an immunosuppressive network [61]. In addition, γδ T cells have an indirect regulatory role in CRC. Activated γδ T17 cells in TME can secrete various cytokines such as IL-17, IL-8, TNF-α, and granulocyte-macrophage colony-stimulating factor (GM-CSF), promoting the further recruitment of immunosuppressive MDSCs. MDSCs are the major regulators of the immune response in various pathological states, and many undifferentiated MDSCs begin to proliferate in response to the stimulation of tumor cells. The massive tumor infiltration of MDSC is closely associated with poor prognosis in various tumors.

As one of the critical members of TME cellular components, tumor-associated macrophages (TAMs) perform an indispensable function in the tumor resistance link and can be recruited to the tumor localization by CCL2, CCL5, and CAFs. TAMs are one of the most infiltrated inflammatory cells in the TME of CRC [62]. Depending on the microenvironment in which TAMs are located, they can be classified as M1 and M2. M1 type TAM mainly secretes pro-inflammatory factors such as IL-1, IL-12, TNF, and CCL, which are involved in inflammatory responses and anti-tumor processes. M2 type TAM mainly participates in wound healing and allergic reactions. It exhibits pro-tumor activity that down-regulates the immunostimulatory factor and up-regulates the immunosuppressive factor [55]. By immunohistochemistry, Yin et al. [63] identified higher levels of TAM infiltration in tumor tissues of 5-Fu-resistant patients. A significant up-regulation of multidrug resistance protein 1 and BCL2 was also detected, which was associated with a poorer prognosis. Mouse macrophages (RAW264.7) co-cultured with mouse CRC cells (CT26.WT) and injected into the peritoneal cavity of mice exhibited reduced sensitivity to 5-Fu.

Adipocytes are a central component of TME, and large-scale data have indicated that obesity is an independent factor contributing to poor CRC prognosis [64]. Ko et al. [65] cultured CRC cells in an adipocyte-rich medium. The results revealed that adipocytes could promote CRC progression by modulating the expression of multiple proteins associated with cancer growth and metastasis. Adipocytes may be involved in the secretion of inflammatory factors in TME. By comparing the levels of pro-inflammatory cells between obese and non-obese CRC patients, it was revealed that the mean levels of IL-6, IL-4, and GM-CSF were significantly higher in obese CRC patients than that in non-obese patients [66, 67]. IL-6 is a critical pro-inflammatory factor mainly derived from tumor cells, CAFs, and TAMs [68–70]. IL-6 modulates CRC cell proliferation, invasive capacity, and sensitivity to chemotherapeutic agents by participating in the regulation of the JAK2/STAT3 axis [63, 71, 72]. It also facilitates the expression of MDR1 and apoptosis inhibitory proteins (Bcl-2, Bcl-xL, and XIAP) and the development of the MDR phenotype in tumor cells [69, 73]. IL-4 is mainly produced by type II helper T (Th2) cells, which enhances tumor growth, metastatic and invasive ability of CRC cells, tumor metabolism, and the growth of metastatic tumors [74]. GM-CSF can serve as an immunostimulatory factor to promote the differentiation and maturation of DCs and macrophages. It also regulates the progression of tumors by inducing the formation of TME [75].

Tumor progression is associated with the formation of organized blood vessels. It is typically facilitated by elevated soluble factors such as VEGFA, promoting the proliferation and angiogenesis of vascular endothelial cells. Pericytes are differentiated from MSCs and colonize the intravascular vessel wall. In addition, CRC cells can also regulate TME through pericytes. Navarro et al. [76] demonstrated that HCT116 co-cultured with pericytes exhibited enhanced migratory and invasive capacity through TGF-β and IGFBP-3, exacerbating the tumor growth in vivo.

### ECM and chemotherapy disorders

Similar to the cells mentioned above, the ECM is responsible for the formation of TME. ECM provides not only mechanical support and protection to tumor cells but also regulates cellular processes, including the proliferation, survival, differentiation, migration, and invasion of cells. As the dominant structural element of the matrix, collagen is the most common and abundant protein and is primarily secreted by CAFs [77]. The content and structural distribution of collagen in tumor tissues indirectly affect the efficacy of drugs [78]. The change of collagen fibers from convoluted to linear morphology around cancer foci affects tumor cell biological behaviors, such as gene expression, cell differentiation, proliferation, migration, and response to drug therapy [77, 79–81]. Collagen fibers can form covalent cross-links with lysine hydroxylase (LOX) and interweave into a stable meshwork structure to resist collagen lysis caused by non-specific protein hydrolases [82]. LOX induces aggregation of integrins and prompts tyrosine autophosphorylation at the FAK397 position. The downstream signaling pathways such as Rho-ROCK, rac, and PI3K–Akt are further activated to stiffen the tumor ECM, preventing the invasion of tumor cells into surrounding tissues and distant metastasis. The infiltration and differentiation of immune cells can also be affected by collagen. The culture of macrophages with type I collagen as a substrate attenuated the ability of macrophages to kill tumor cells, indicating that type I collagen inhibits the differentiation of TAMs toward the M1 phenotype [83]. The formation of blood vessels in the tumor stroma also requires the involvement of collagen [84]. The synthesis and deposition of basement membrane collagen (especially type IV) is essential for tumor angiogenesis, and the inhibition of collagen metabolism has been demonstrated to have anti-tumor angiogenic effects. It has been revealed that type IV collagen not only regulates (promotes or inhibits) the growth and proliferation of vascular endothelial cells but also stimulates their adhesion and migration [85].

LN is a valuable component of the ECM in the basement membrane with critical functions in tumor invasion. It is a multi-structured heterodimeric protein consisting of an α, a β, and a γ chain [86]. Unlike most ECM proteins, LNs have a degree of tissue and temporal specificity [87]. They are significantly associated with cancer cell survival and proliferation, angiogenesis, migration, and destruction of basement membranes, cancer progression, and metastasis to distant organs [88–90]. For example, LN521 can promote proliferation and invasion of CRC cells by enhancing STAT3 phosphorylation [91].

PGs help maintain the morphology of the ECM and consist of one or more GAGs covalently linked to a core protein. They are widely distributed in the ECM, cell surface, and intracellular secretory granules. Hyaluronan (HA) is one of the common GAGs. Its synthesis is particularly robust in developing and trauma-repaired tissues, promoting cell migration and proliferation. The biological activity of HA depends on its molecular weight and the receptors interacting with it, including CD44, lymphatic endothelial receptor (LYVE-1), and HA endocytic receptor (HARE). These receptors maintain the endocytic environment in normal tissues and inhibit cell proliferation and spreading [92, 93]. In normal tissues, HA maintains a stable microenvironment and inhibits cell proliferation and migration, while low molecular HA cleaved by hyaluronidase or reactive oxygen species (ROS) can promote immune cell aggregation [94]. The acetyl heparan sulfate proteoglycan Syndecan-1 (Sdc-1) has a pivotal role in maintaining cell morphogenesis, promoting tissue repair, and regulating immune function. In normal conditions, Sdc-1 is anchored to the cell membrane surface, where CRC cells can secrete MMP7 to hydrolyze the anchored Sdc-1 and release it into the blood [95]. Through binding to EGFR to activate the downstream RAS/RAF/MEK/ERK signaling pathway, Sdc-1 promotes the acceleration of CRC cells from the chemosensitive G1 phase to the chemoresistant S phase [96, 97]. It also maintains tumor cell stemness by regulating the Wnt/IL-6/STAT3 signaling pathway and induces an MDR phenotype in tumor stem cells [98].

The collagens, PGs, GAGs, and LNs mentioned above, along with elastin and FN, form a highly fibrotic ECM that is distinct from normal tissue. Various cytokines and growth factors secreted by tumor cells and stromal cells promote the continuous degradation and deposition of ECM components, leading to a substantial increase in stiffness, which in turn prevents the entry of chemotherapeutic agents into tumor cells. Appropriate DDS could assist chemotherapeutic drugs in crossing the solid tumor barrier to target cells.

### The special biochemical environment of TME and chemotherapy obstacles

The interaction of tumor cells, stromal cells, and ECM-related components mentioned above result in a tumor TME signature completely different from that of normal tissue. Abnormal supply vasculature and the accompanying tissue hypoxia are the distinguishing features of TME. Similar to the physical barrier of rigid ECM preventing drug entry, hypoxia and vascular reduce the efficacy of chemotherapeutic drugs.

Hypoxia is a common feature of TME in tumor tissues, and there are multiple causes of hypoxia. The first one is that the rapid proliferation of tumor cells consumes a large amount of oxygen. Tumor cells induce hypoxia through various mechanisms, such as high metabolic rate and high oxygen consumption. These mechanisms lead to endothelial dysfunction or disrupt oxygen delivery due to various effects on blood vessels, creating a chronic hypoxic environment. The diffusion of oxygen is impeded by the massive connective tissue proliferation, resulting in a strongly fibrotic ECM. In hypoxic conditions, tumor cells secrete various vascular growth factors to promote the formation of abnormal blood vessels. The invasion and metastatic ability of tumor cells are further enhanced. It has a selective effect on tumor cells, in which highly malignant tumor cells survive in the hypoxic microenvironment, resulting in the insensitivity of tumor cells to chemotherapeutic drugs or radiation therapy [99]. The hypoxic response of tumor cells is primarily driven by the hypoxia-inducible factor (HIF) [100]. In addition, the low O_2_ level also limits proline hydroxylation of HIF1 under hypoxia, resulting in increased HIF1 protein levels [100, 101]. HIF-mediated pathways affect tumor proliferation and differentiation through mTOR signaling, erythropoiesis, angiogenesis, cell growth, and differentiation [102]. Necrosis often develops in hypoxic regions where tumor spread and metastasis are more likely to occur. HIF-1α performs a pivotal function in tumor cell invasion, metastasis, immortalization, and tumor angiogenesis. HIF-1α directly or indirectly regulates the expression of transcription factors that control the EMT process, including Notch [103], VEGF [104, 105], platelet-derived growth factor (PDGF) [106], TGF-β [107], and ZEB1 [108]. HIF is closely associated with chemoresistance by activating the β-catenin/Wnt signaling pathway maintaining tumor cell stemness [109]. Tang et al. [110] revealed that a hypoxic culture environment promotes STAT3 phosphorylation in CRC cells and promotes tumor progression through PI3K/AKT pathway activation.

HIF alleviates tissue hypoxia by inducing the expression of multiple pro-angiogenic factors, including VEGF, angiopoietin-2 (Ang-2), PDGF-β, inducible nitric oxide synthase 2 (iNOS2), and endothelin-1 (ET-1), thus creating additional vasculature for nutrient delivery [111, 112]. Anti-angiogenic targeted drugs are applied in tumor therapy as well. For example, bevacizumab is suitable for metastatic CRC, advanced non-small cell lung cancer (NSCLC), and hepatocellular carcinoma (HCC) [113]; regorafenib can be employed to treat metastatic CRC [114]; sorafenib has a dual anti-tumor effect of inhibiting tumor cell growth and tumor tissue angiogenesis [115]. The hastily formed tumor blood vessels are distinguished from general blood vessels by their uneven distribution, large capillary spacing, incomplete endothelial cells, and disrupted basement membrane. These problems lead to fragile vascular walls and vascular hyperpermeability, leading to interstitial tumor hypertension [116, 117]. The abnormal vascular structure and the consequent high pressure in the tumor interstitium result in inadequate perfusion of the tumor tissue, hindering the penetration of chemotherapeutic agents.

In addition to the obstacles mentioned above, the utilization of glycolysis by tumor cells for energy supply and the high amounts of lactic acid production lead to a decrease in pH in tumor tissues, posing a challenge to the study of appropriate DDSs for CRC chemotherapy [118]. Most of the current therapeutic drugs are alkaline and cannot easily penetrate into cells after protonation of the acidic TME, resulting in natural drug resistance in tumor cells. The increased activity of hydrogen ion transporters in tumor cells makes it difficult for chemotherapeutic drugs to induce intracellular acidification and cause apoptosis of tumor cells [119].

All these obstacles require us to employ more appropriate DDSs to overcome the heavy obstacle of TME and realize the effect of chemotherapeutic drugs.

## Targeted NPs in CRC research

**Fig. 2:**
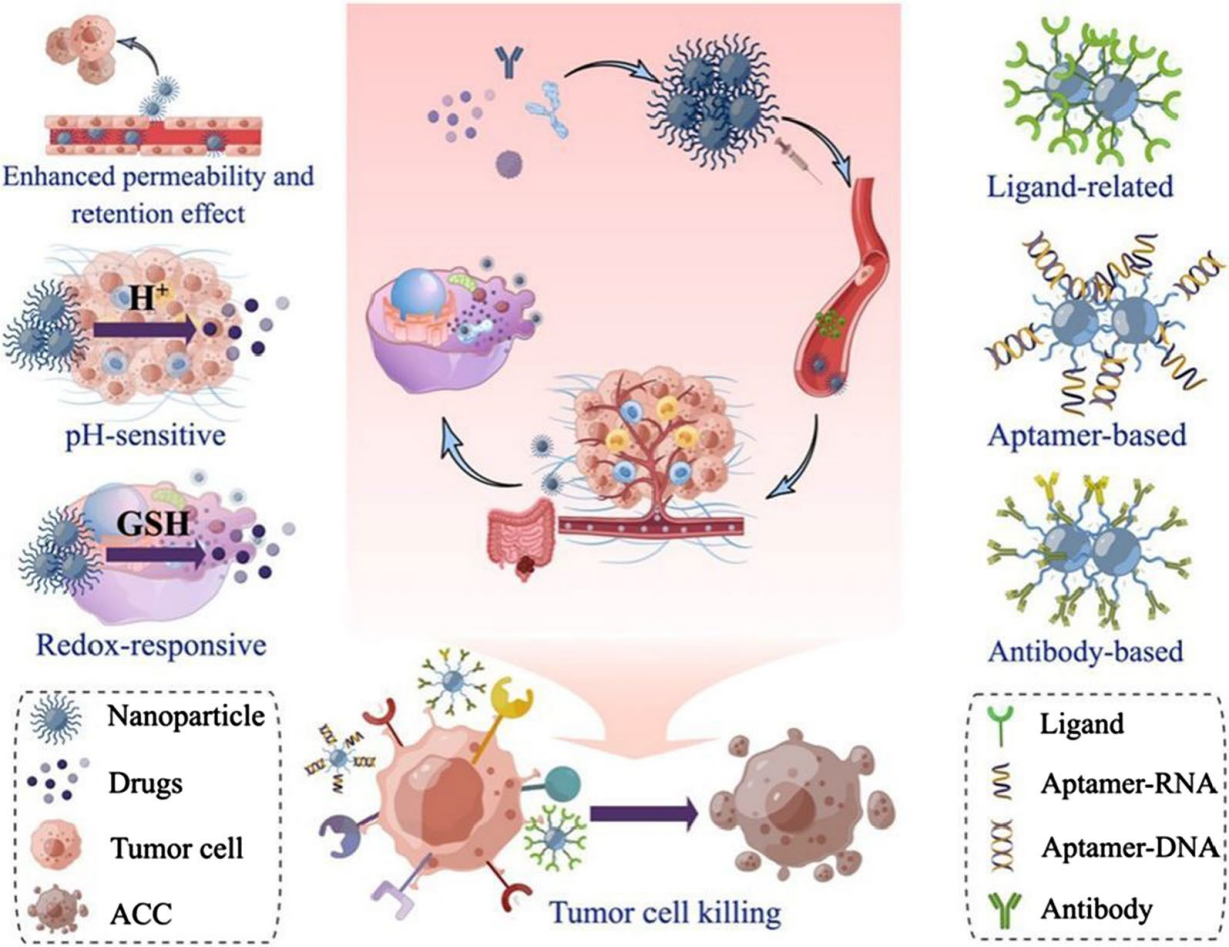
Classification and mechanisms of targeting nanoparticles

In order to achieve more effective targeting of chemotherapeutic drugs to tumor cells, thus specifically killing them and reducing drug toxicities to benefit more patients, NPs have gained the attention of researchers as a new class of DDSs (Fig. 2). Compared to free drugs, nano-drug delivery systems exhibit improved bioavailability, enhanced tissue targeting, reduced off-target adverse effects, improved bioavailability, and greater in vivo stability [120]. With their unique advantages, NPs have been employed to diagnose and treat various diseases, such as ocular disease [121], inflammatory bowel disease [122], osteoporosis [123], Alzheimer’s disease [124], and stroke [125]. In tumor treatment, chemotherapy drugs can be a systemic treatment for primary lesions and metastases, but the limitations are evident. Most chemotherapeutic drugs have poor selectivity, making them difficult to break through the heavy obstacle of TME to reach tumor cells. Furthermore, a high dose of these chemotherapeutic agents can lead to side effects.

Targeted NPs can be divided into two categories based on substrate composition: naturally-derived NPs and synthetic NPs. Furthermore, naturally-derived NPs contain biological components or cell-derived vesicles, and synthetic NPs are classified into inorganic and polymeric NPs. Compared to other carriers, drug delivery employing targeted NPs enhances permeability and EPR effects, with higher transport efficiency across cell membranes. Nanocarriers need to be biocompatible and biodegradable, with less impact on cell growth and metabolism [126]. Targeted NPs can be loaded with extensive active substances, such as anti-tumor drugs, siRNA, proteins, and contrast agents. In the wake of the rapid advancement of nanobiotechnology, anti-tumor nano-drugs are expected to improve cancer treatment strategies and enhance therapeutic efficacy [127]. Targeted NPs as DDSs of chemotherapeutic drugs can improve the specificity of tumor tissues by altering pharmacokinetics and tissue distribution, inhibiting tumor growth, and reducing the drug toxicity to normal tissues [128]. In addition, the target molecule modification on the external surface of NPs can improve the drug concentration in tumor tissues and realize the effective treatment of tumors and the integration of tumor diagnosis and treatment. Targeted NPs can achieve tumor-targeted delivery and intra-tumor penetrating transport of chemotherapeutic drugs, avoid non-specific systemic release of drugs, improve pharmacokinetic and pharmacodynamic properties of drugs, and assist drugs in overcoming tumor cell resistance mechanisms [129]. Extensively studied polymer-targeted NPs for cancer chemotherapy can protect the contents from circumstance and provide a sustained and tunable release rate through a targeting strategy. The raw materials for the synthesis of targeted NPs include natural polymers (e.g., chitosan, gelatin, alginate, HA, and albumin) and synthetic polymers [e.g., polyethylene glycol (PEG), polylactide-ethyl lactide (PLGA), polylactic acid (PLA), and polycaprolactone (PCL)]. Despite the complicated synthesis of natural polymers, they have promising bioavailability and biodegradability. On the other hand, synthetic polymers are widely available and easy for synthesis and surface modification, while their biodegradability is less favorable [130].

The main benefit of targeted NPs as carriers for chemotherapeutic drugs is the targeting effect. The mechanism of targeting tumor tissues can be classified into passive targeting and active targeting. Passive targeting enhances the EPR effect by exploiting the basement membrane incomplete vascular system in tumor TME against the tumor tissue. It can also utilize the special pH, enzyme environment, and intracellular reducing environment of the tumor site to achieve drug release at specific sites for targeted drug delivery. Passive targeting depends mostly on the size effect of the drug or its carrier. Active targeting is achieved by functionalized modification on the surface of NP carriers. Probe molecules that can bind specifically to the target molecule, such as antibodies, peptides, sugar chains, and nucleic acid aptamers, are coupled to the carrier surface by chemical or physical methods. The proportion of nano-drugs reaching the target can be increased using ligands to bind to tumor cells or tumor TME overexpressed receptors. For example, Govindarasu et al. [131] prepared PLGA NPs loaded with kaempferitrin and bound folic acid (FA) on the surface of the NPs. Due to the significantly higher expression of FA receptors in tumor tissues, the killing effect of kaempferitrin on CRC cells could be enhanced by the targeted binding of FA and its receptors. Targeted NPs can allow improved targeting of tumor tissues and tumor cells, reducing the hindrance to chemotherapeutic drug application caused by stiff ECM and non-specific tissue damage.

The design of suitable targeted NPs as DDSs for chemotherapeutic agents requires the following considerations: (a) favorable biocompatibility and degradability; (b) superior stability of physical and chemical properties; (c) appropriate size for the aggregation of NPs at the tumor site through imperfect tumor vasculature; (d) modification of NPs for the aggregation in the specific pH and enzymatic environment of the tumor site. Based on the above factors, heavily designed targeted NPs have been studied extensively in CRC chemotherapy. This review briefly summarizes them and classifies them into the following categories: facile NPs; pH-sensitive targeted NPs; redox-responsive targeted NPs; ligand-related active targeted NPs; aptamer-based targeted NPs; antibody-based targeted NPs; antibody fragment-based targeted NPs.

### Passive targeted NPs

#### Facile NPs

**Fig. 3:**
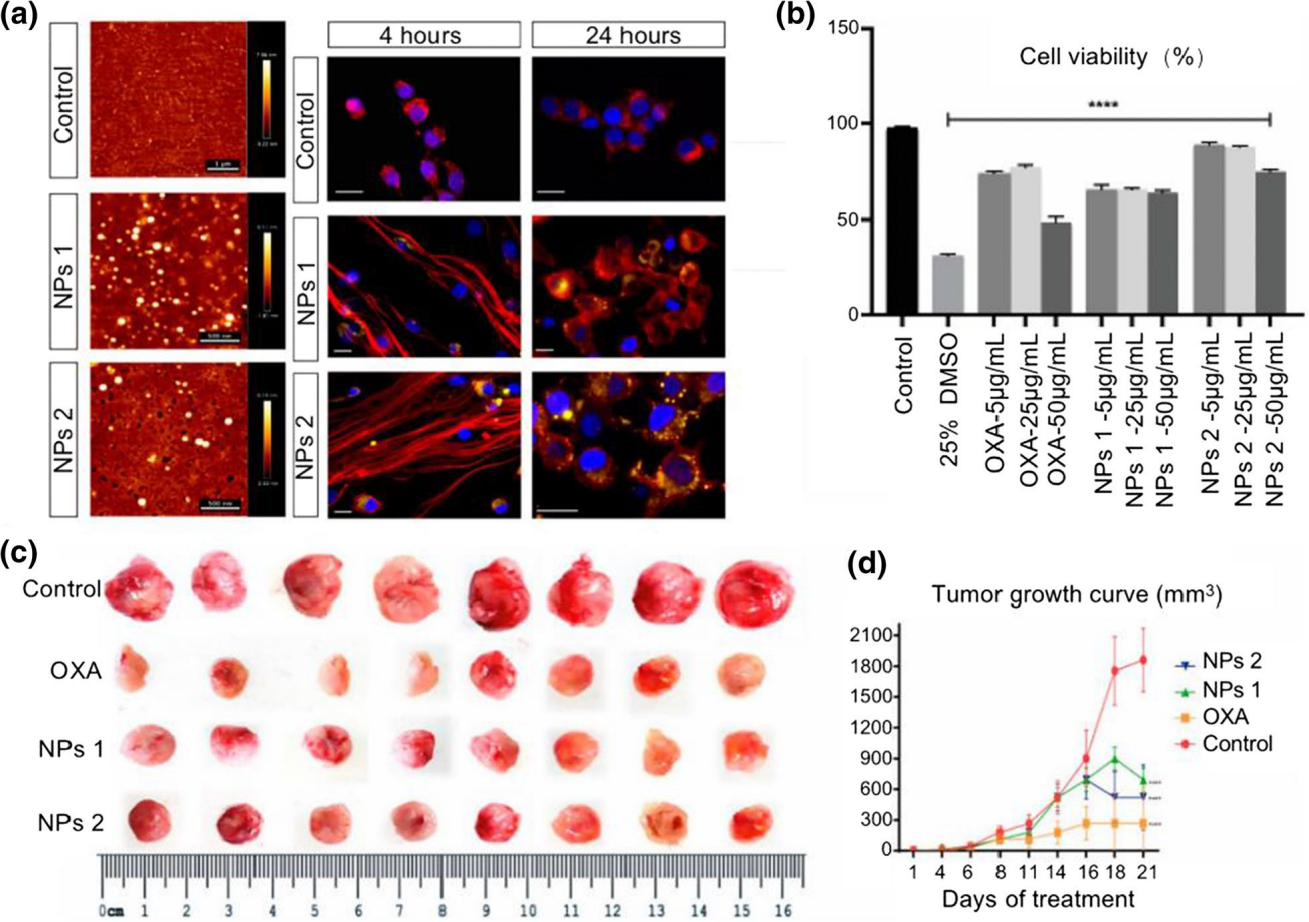
Effects of oxaliplatin and retinoic acid loaded in cholesterol-coated PLGA NPs on tumor cells. **a** AFM images of Control group, NPs 1 group and NPs 2 group; fluorescence images display the internalization of NPs by CT26 cells, with monitored NPs in yellow; **b** Cell viability of SW-480 cells after treatment with different concentrations of OXA and PLGA NPs for 24 h; **c** Changes in tumor volume after treatment with different concentrations of OXA and PLGA NPs in a subcutaneous transplantation tumor model in mice; **d** Tumor growth curves for each group were compared with the negative control group (Reproduced with permission from [133]. © 2020 by *Ana Luiza C. de S. L. Oliveira et al.)*

Facile NPs passively deliver chemotherapeutic agents to tumor tissues through enhanced EPR effects. In normal tissues, the microvascular endothelial gap is dense and structurally intact, leaving no potential for macromolecules and lipid particles to easily cross the vascular wall. In contrast, tumor tissues are rich in blood vessels with a wide vascular wall gap, poor structural integrity, and missing lymphatic return flow. Due to the EPR effect, nano-drugs with diameters between 10 and 100 nm are enriched in tumor tissues, forming a passive targeting of nano-drugs to tumor tissues [132]. Common natural polymer synthetics include albumin and chitosan, and synthetic polymers include PEG and PLGA. PLGA is a degradable synthetic polymeric organic compound with promising biocompatibility, non-toxicity, and excellent capsule- and film-forming properties. It is widely employed in pharmaceuticals, medical engineering materials, and modern industries. PLGA-NPs can encapsulate hydrophobic chemotherapeutic drugs, improving their water solubility and targeting ability. Oliveira et al. prepared PLGA-NPs using an emulsified solvent extraction/evaporation method for encapsulation and targeted delivery of oxaliplatin and retinoic acid to tumor sites. The PLGA-NPs were coated with cholesterol to enhance endocytosis of the composite NPs by tumor cells. NPs loaded with oxaliplatin exhibited enhanced pro-tumor cell apoptosis and protection against non-tumor cells compared to free oxaliplatin (Fig. 3) [133]. In addition to solution extraction methods, microfluidics allows for more precise control of drug release. Ghasemi Toudeshkchouei et al. [134] prepared PLGA-NPs loaded with 5-FU at an average diameter of about 119 nm using microfluidics, facilitating the control of drug release and reducing adverse effects due to drug dose. The negative charge on the surface of PLGA-NPs decreases the rate of cellular internalization, while functionalization of the NP surface with cationic polymers can significantly enhance the cellular uptake and aggregation of NPs at tumor sites. Xiao et al. [135] exploited chitosan to surface-functionalize PLGA-NPs loaded with camptothecin and curcumin. The chitosan-functionalized PLGA-NPs with positive surface charge significantly improved the uptake of tumor cells and increased intracellular drug concentration.

**Fig. 4:**
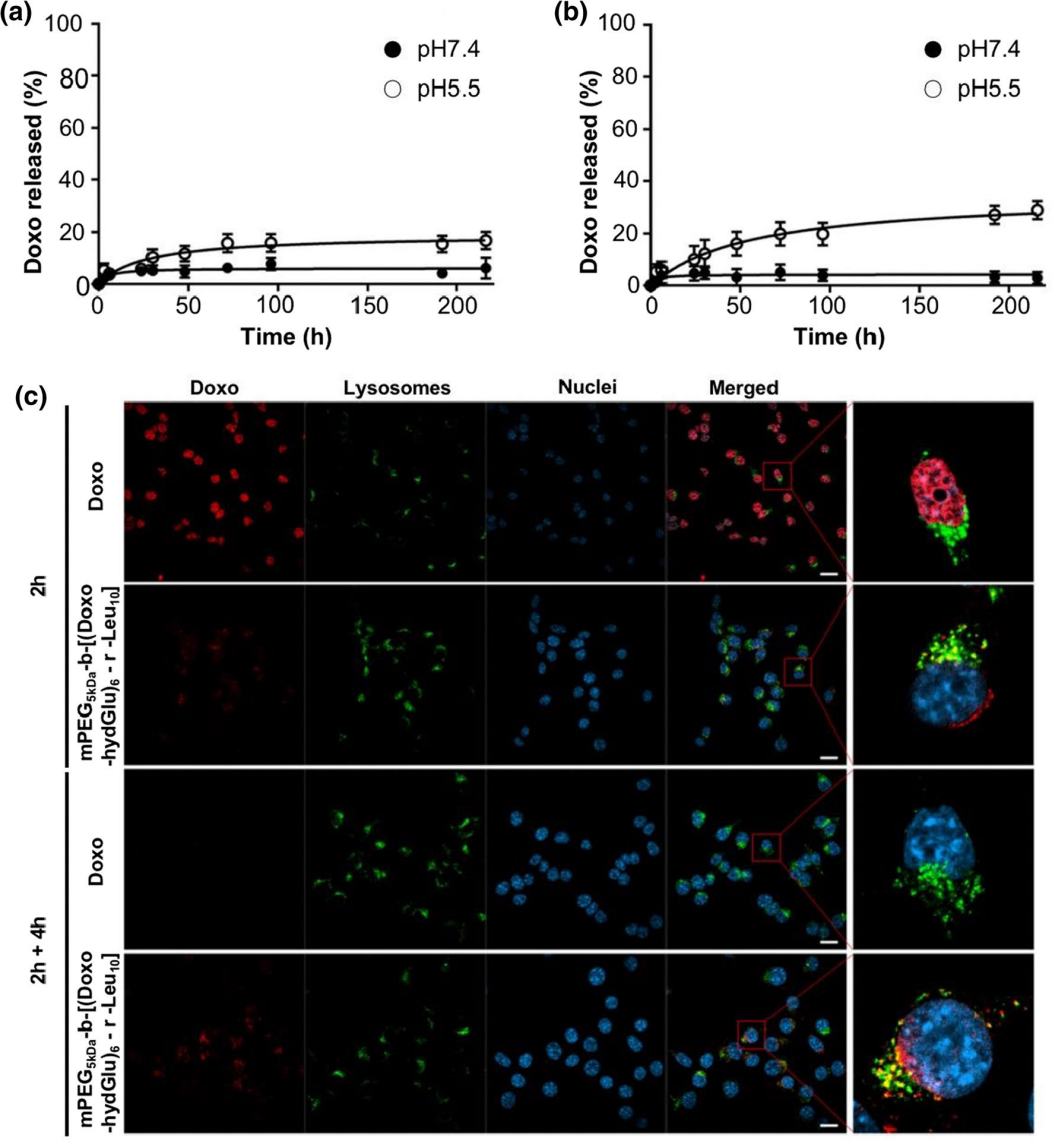
pH-sensitive hydrazone bonds attached doxorubicin to mPEG-based diblock copolymers. **a** Rates of Doxo release from different ratios of Leu and Glu copolymers bound to mPEG at varying pH conditions; Doxo release profile of mPEG_5kDa_-(Doxo-hydGlu)_16_; **b** Doxo release profile of mPEG_5kDa_-b-[(Dox-hydGlu)_6_-r-Leu_10_]; **c** Confocal microscopy images of CT26 cells and nanodrug after 2 h incubation and a further 4 h incubation. Nanodrug delivery into the cells and cleavage of the hydrazone bond in an acidic lysosomal environment resulted in Doxo release (Reproduced with permission from [146]. © 2021 Elsevier B.V. All rights reserved)

Other therapeutic components such as pigment epithelium-derived factor (PEDF) [136], adriamycin (ADR) [137], and nucleic acid molecules [138, 139] can be prevented from aggregation and phagocytosis by facile NPs, prolonging their circulation time in vivo and passively targeting tumor tissue through enhanced EPR effects. Facile NPs can reach the target sites through incomplete neovascularization of tumor tissue and stiff ECM, reducing non-specific tissue damage and overcoming the barriers to chemotherapy caused by stiff ECM. Passive targeting through EPR effects is less selective and cannot specifically target tumor cells in TME, failing to effectively enhance the ability of tumor cells to uptake NPs for better efficacy (Fig. 4).

#### pH-sensitive targeted NPs

As mentioned above, hypoxia and acidic TME contribute to chemotherapy resistance. Smart delivery systems prepared with pH-sensitive polymers perform stably in physiological environments and release drugs in tumor tissues with reduced pH, resulting in targeted anti-tumor efficacy and reduced side effects. The pH-responsive polymers with basic residues or ionizable acidic residues are susceptible to ionization by changes in the pH of the surrounding medium. In addition, they are specifically triggered by environmental pH, resulting in changes in physicochemical properties (e.g., solubility, chain conformation, surface activity, and conformation) [140]. Polymers containing ionizable weak acid groups and acid-sensitive linking segments (e.g., imine bonds, tertiary amine bonds, amide bonds) have significantly different physicochemical properties upon pH changes. They have been widely employed in the manufacture of acidic TME-responsive cancer nanotherapeutics. Organic acid polymers with weak acid groups alter their ionic strength with the pH of the medium. As a drug carrier, polyacrylic acid (PAA) has carboxyl groups that hydrolyze and break in acidic TME, causing the polymeric NPs to rupture and release the anti-tumor agents [141]. Lee et al. [142] exploited poly (acrylic acid-*co*-methyl methacrylate) copolymer loaded with cisplatin to perform well-targeted therapeutic effects in the CT26 mouse CRC model. The amine bond is easily hydrolyzed and unstable under acidic conditions. Based on their special pH-responsive properties, pH-sensitive NPs were constructed for CRC treatment. Zhang et al. [143] constructed TME pH-sensitive cleavable mPEG 2k-DOX by grafting doxorubicin (DOX) onto imine bond-based aldehyde HA and further binding it to mPEG. Compared with the free drug, the pH-sensitive loading of NPs significantly increased the long circulation time of doxorubicin in vivo by about 12.5-fold and effectively targeted tumor tissues to reduce toxicity. Feng et al. [144] have synthesized nanomicelles based on PEG and poly (*N*-(*N*ʹ,*N*ʹ-diisopropylaminoethyl) aspartamide) (P(Asp-DIP)) and poly (lysine-cholic acid) (P(Lys-Ca)) of nanomicelles. The tertiary amino group in P(Asp-DIP) is pH-responsive and releases drug components by a hydrophobic–hydrophilic transition in acidic TME. Encapsulation of paclitaxel and superparamagnetic iron oxide in copolymers (SPIO) as MRI-visible drug delivery systems demonstrated that paclitaxel was delivered to tumor tissue by pH-sensitive micellar NPs. Boronic ester bonds [145] and hydrazone bonds [146] are considered unstable under acidic conditions and commonly employed in preparing pH-sensitive NPs. Brunato et al. prepared amphiphilic diblock copolymers based on mPEG and polyamino acid blocks and bound Doxorubicin to the diblock copolymers through pH-sensitive hydrazone bonds and demonstrated a significant increase in drug release rate in an acidic environment [146]. Most alkaline chemotherapeutic drugs cannot readily penetrate cells after protonation in acidic TME, leading to the natural resistance of tumor cells. The pH-sensitive targeted NPs could release drugs to kill tumor cells in acidic hypoxic TME, which also reduces non-specific tissue damage.

#### Redox-responsive targeted NPs

**Fig. 5:**
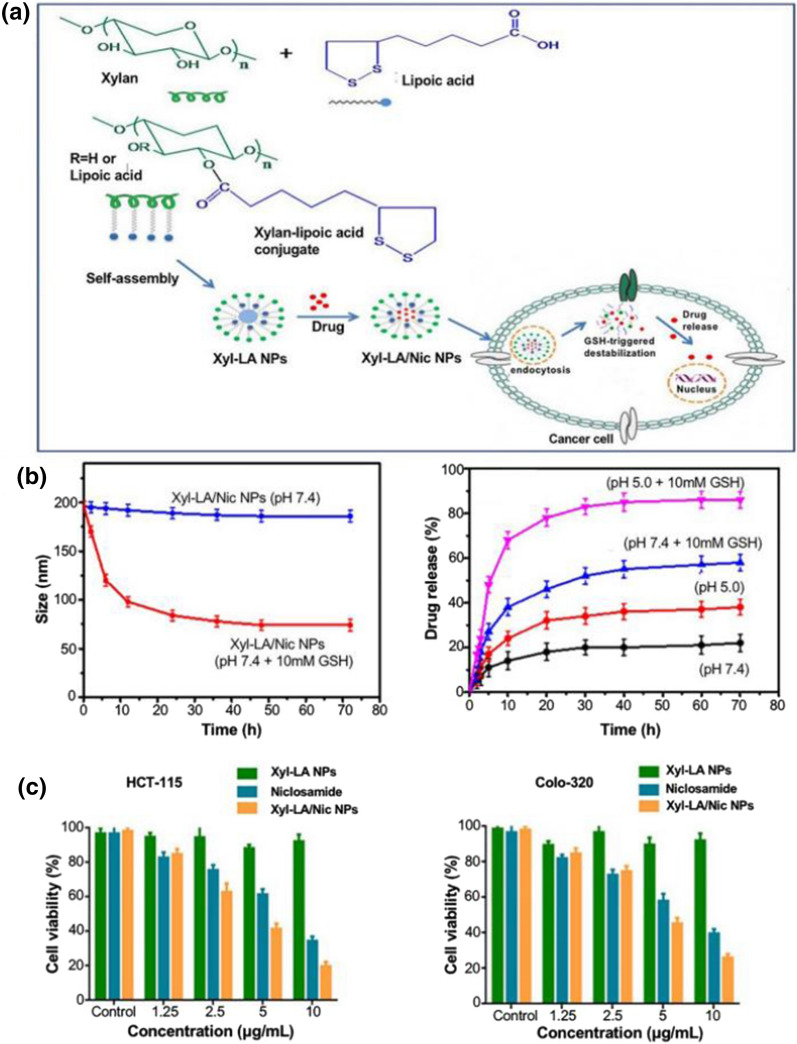
Redox-responsive NPs prepared on the basis of xylan-lipoic acid for the delivery of niclosamide for the treatment of CRC. **a** Schematic diagram of the preparation of Xyl-LA/Nic NPs; **b** stability and degradation of Xyl-LA/Nic NPs at different pH; **c** drug release rate of Xyl-LA/Nic NPs at different pH and in the presence or absence of GSH (Reproduced with permission from [150]. © 2020 Elsevier Ltd)

TME redox status differed significantly from the microenvironment of normal tissues and cells. Glutathione-related metabolic enzymes and ROS are hyper-expressed in different subcellular structures, resulting in an imbalance of redox status [147]. The high amount of ROS and high concentration of the reducing substance glutathione (GSH) in tumor cells lead to an overall oxidative stress state. Redox-sensitive NPs can be prepared using the TME-specific redox microenvironment with GSH and ROS as stimulators of intelligent response NPs [148]. GSH is pivotal in regulating intracellular redox homeostasis and is closely associated with cancer development, progression, and metastasis [149]. Disulfide bonds are the most common reduction-responsive chemical bonds connecting NPs and anti-tumor drugs, which can be reductively disrupted in GSH-rich TME and then control drug release. Sauraj et al. [150] prepared NPs conjugated with lipoic acid (LA) and xylan (Xyl) for the loading of niclosamide (Nic). Xyl-LA/Nic NPs performed steadily under physiological conditions and released the drug rapidly in the presence of GSH. Thioether bonds can also be used to prepare redox-responsive NPs, which break rapidly in tumor cells to release loaded drugs (Fig. 5). Wang et al. [151] coupled SN-38 to ethylene glycol oligomers (OEG) via thioether bonds to form redox-responsive NPs. The results suggested that thioether bonds are responsive to both ROS and GSH. The thioether bond can be sulfated in the presence of GSH or hydrolyzed under the oxidative influence of ROS, both of which can help redox-responsive NPs release drugs to tumor sites. Diselenium bonds have lower bond energies, which are more sensitive than sulfur-containing counterparts under mild stimulation conditions. They also effectively target drug release in response to changes in redox levels in the microenvironment [152]. Redox-responsive targeted NPs, besides reaching tumor cells through the stiff ECM with enhanced EPR effect as facile NPs, are activated by the specific redox environment of tumor tissue to release drugs, which significantly reduces non-specific tissue damage.

### Active targeted NPs

#### Ligand-related active targeted NPs

**Fig. 6:**
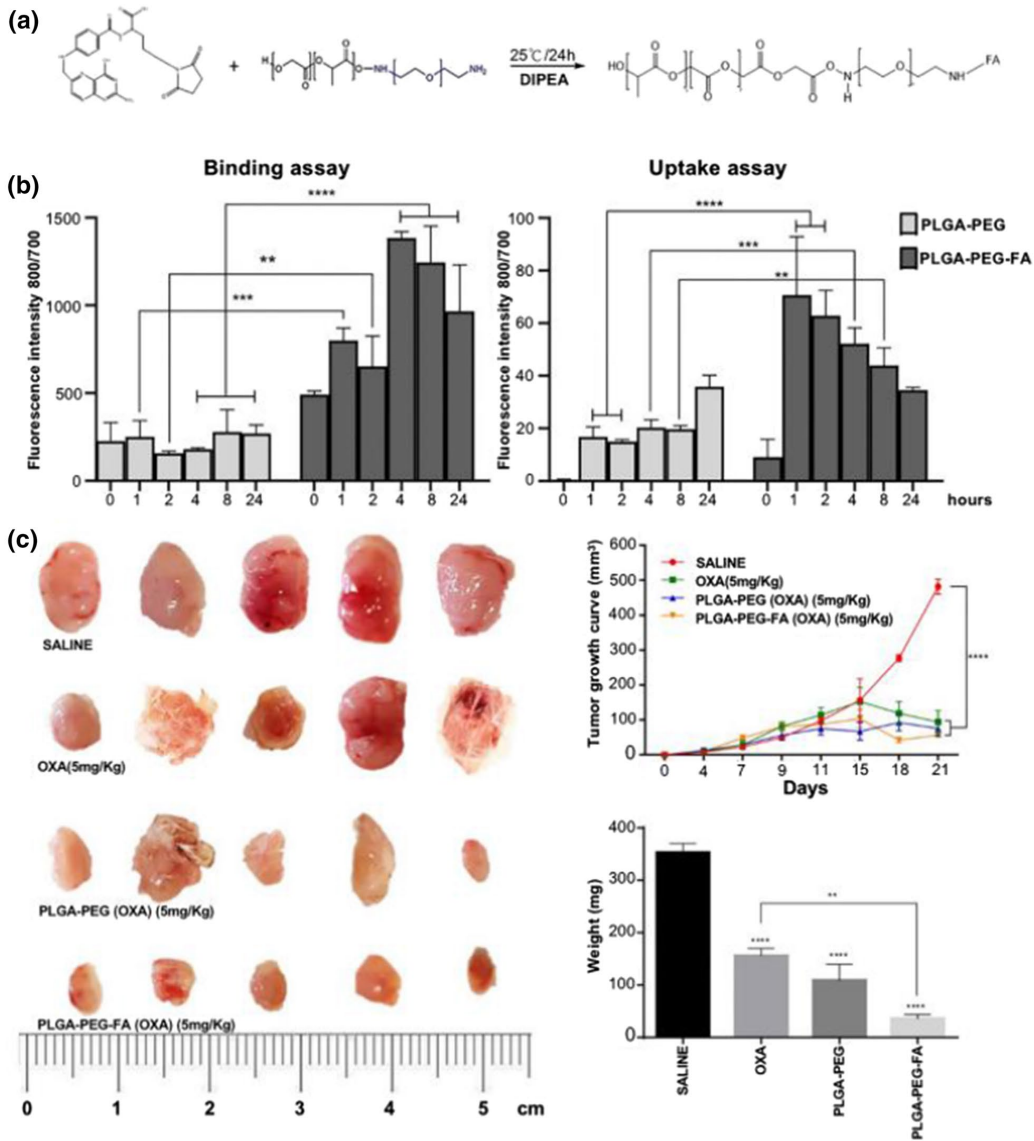
Folic acid modification of PLGA NPs containing oxaliplatin enhances its targeting and antitumor capacity. **a** Schematic of the formulation of PLGA-PEG-FA NPs; **b**: analysis of cell binding and uptake of NPs under fluorescence microscopy after incubation of PLGA-PEG and PLGA-PEG-FA NPs on CT26 cells for different times; **c** changes in tumor volume and weight of CT26 tumor-bearing mice under different treatment groups (Reproduced with permission from [156]. © 2021 by *Ana Luiza C. de S.L.Oliveira et al.)*

By chemical or physical methods, ligand-based active targeting of NPs specifically binds probe molecules (e.g., antibodies, peptides, glycoconjugates, and nucleic acid aptamers) to target molecules on the surface of NPs. The high expression of transferrin receptor (TfR) is commonly associated with rapidly proliferating cells. It is up-regulated in various tumor cells and is available as targeted therapy. Durán-Lobato et al. [153] used diamine bonds to attach Tf to the surface of PLGA NPs and loaded Δ9-tetrahydrocannabinol, effectively improving the bioavailability, reducing side effects, and targeting Tf-high expressing tumor cells for action. Compared to proteins, Tf-binding peptides with short sequences are readily modifiable and are more suitable for targeted nanomedicine development. Tf-binding peptide-modified polymers deliver doxorubicin targeting to HCT 116 cells and effectively inhibit tumor progression in vivo [154]. Folate receptors (FRs) are extensively expressed on the surface of tumor cells but marginally expressed in normal cells. By targeting folate receptors, NPs modified with folate acid (FA) can concentrate on the surface of tumor cells with high expression of folate receptors to increase drug uptake of tumor cells and achieve targeted tumor therapy. Layer-by-layer deposition of FA and raltitrexed on the surface of polystyrene NPs significantly increased the uptake of tumor cells [155]. During in vivo experiments, FA-modified oxaliplatin-loaded PLGA NPs remarkably reduced tumor load and decreased drug resistance (Fig. 6) [156]. HA has multiple receptors overexpressed on the surface of tumor cells and enables targeted drug delivery to tumors based on a ligand–receptor binding mechanism, with excellent biocompatibility and degradability. CD44 is a transmembrane glycoprotein that is overexpressed on the surface of many tumor cells. The highly specific binding of CD44 and HA and the favorable biocompatibility of HA are essential for the design and preparation of HA-mediated tumor-targeting drugs. The active targeting of HA and the promising drug encapsulation properties of PEI can be used to facilitate the effective delivery of sequence-specific double-stranded RNA (dsRNA) to tumor tissues and promote endocytosis [157]. The competitive binding of HA oligosaccharides (oHA) to CD44 could reverse HA-induced chemoresistance in CRC [92]. Easily accessible and manipulated short peptide sequences are also widely applied in the study of ligand-based active targeted NPs. The tumor-penetrating peptide RGD recognizes integrin αvβ3 receptors overexpressed by tumor vascular endothelial cells, thus targeting therapeutic effector molecules to the tumor site. Zhong et al. [158] prepared paclitaxel-loaded PLGA-NPs with functionalized surface modified with iRGD. PLGA-NPs passively target tumor tissue through prolonged in vivo circulation time and enhanced EPR effect. The iRGD on their surface actively recognizes tumor vascular endothelial cells that highly express integrin receptors, allowing PLGA-NPs to break down tissue barriers. Dual modification of the surface of PLGA-NPs by iRGD and EGFR single-domain antibodies demonstrated enhanced targeting ability both in vitro and in vivo [159]. In addition, tumor vascular endothelium-targeting peptides (NGR) [160], TME-targeting ligands [161], and peptide VATANST [162] are also employed in the development of ligand-based active targeted NPs.

#### Aptamer-based targeted NPs

**Fig. 7:**
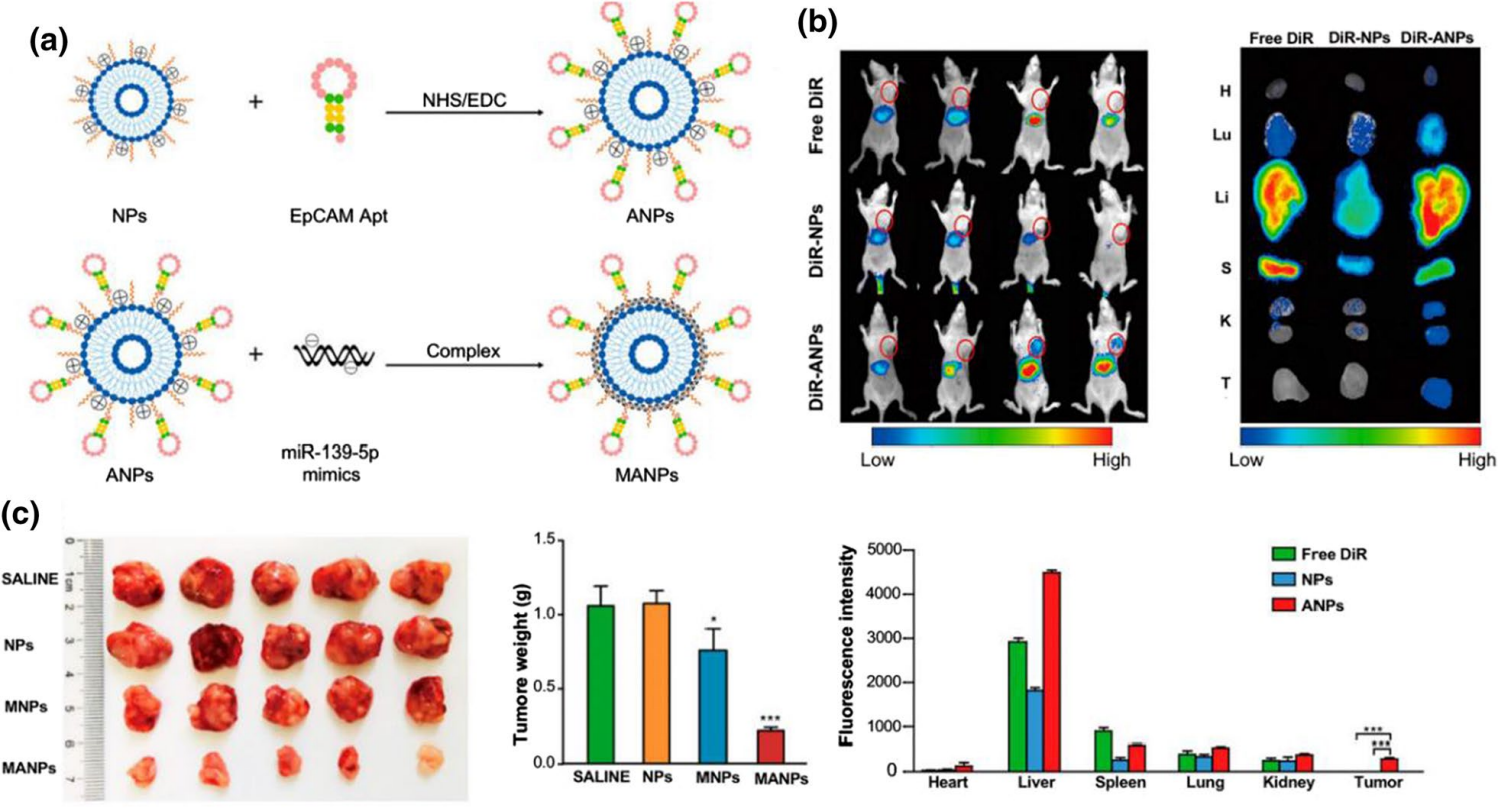
EpCAM aptamer-functionalized cationic liposome NPs loaded with miR-139-5p inhibited CRC cells. **a** Schematic diagram of the preparation method of NPs and ANPs; **b** fluorescence images of HCT116 subcutaneously transplanted tumor-bearing mice after tail vein injection of free DiR, DiR-NPs and DiR-ANPs, revealing the in vivo targeting and distribution of ANPs; **c** tumor volume changes after group treatment in HCT8 tumor-bearing mice (Reproduced with permission from [168]. © 2019 American Chemical Society)

The aptamer is a single-stranded oligonucleotide molecule that strongly binds to the specific target molecule with high affinity. The advantages of aptamer are as follows: high affinity; convenient to obtain, synthesize, and stable; non-immunogenic and reusable. The aptamer as a targeting ligand can be applied to the surface of drug carriers as a targeting moiety to deliver targeted chemotherapeutic drugs, providing a feasible way to improve the clinical efficacy of drugs [163]. AS1411 is a guanosine-rich oligonucleotide that exerts anti-cancer activity by binding to nucleoproteins on the cell surface, leading to S-phase cell cycle arrest and the growth inhibition of various solid tumors [164]. Yu et al. [165] used AS1411 as the aptamer to functionalize paclitaxel-loaded albumin NPs. The results demonstrated that increased uptake of AS1411-modified drug-loaded particles by tumor cells significantly enhanced the anti-tumor activity of paclitaxel while reducing systemic toxicity. Epithelial cell adhesion molecule (EpCAM) is a transmembrane glycoprotein that plays an important role in cell signaling, proliferation, differentiation, organ formation, and maintenance in vitro and in vivo [166]. The differential expression of EpCAM in normal and CRC cells makes it an ideal therapeutic target. Zhang et al. [167] used EpCAM aptamers and PEG NPs to target and deliver tanshinone II-A to tumor sites. Compared with free drugs, targeted NPs can effectively improve the bioavailability and targeting ability of the tanshinone II-A, inhibiting tumor proliferation and metastasis. EpCAM aptamer-modified PEG-cationic liposomal NPs loaded with oncogenic miR-139-5p mimics exert anti-tumor effects through electrostatic adsorption of miR-139-5p mimics, long circulation time in vivo with the help of PEG and targeted binding of EpCAM to CRC cells (Fig. 7) [168]. Aptamer-based NPs with high drug loading capacity and long circulation time in vivo show exciting results, especially in tumor therapy, which is a hot topic for future research. Due to the short half-life of the aptamer and its susceptibility to degradation, aptamer-based targeted NPs can be considered as carriers of chemotherapeutic drugs.

#### Antibody-based targeted NPs

**Fig. 8:**
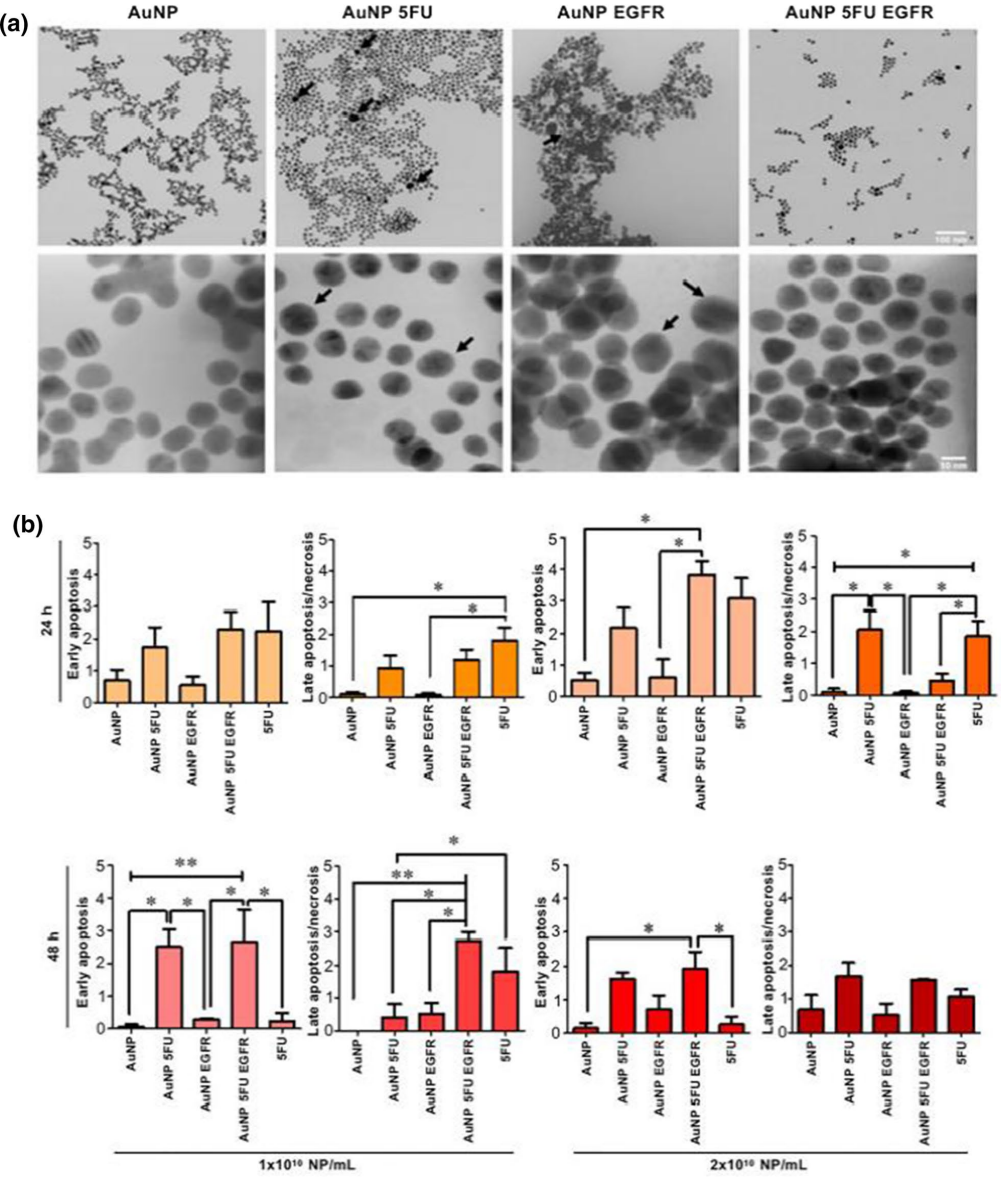
Anti-EGFR-coated AuNPs target 5-Fu delivery to CRC cells. **a** Morphological and physical characterization of AuNPs by transmission electron microscopy; **b** toxicity of different concentrations of NPs on HT-29 cells after treatment for 24 and 48 h, respectively (Reproduced with permission from [170]. © 2020 by *Raquel B. Liszbinski et al.)*

The strengths of antibodies as targeting molecules are their ease of manufacture and modification, high tissue penetration, excellent stability, and superior specificity and affinity [169]. Monoclonal antibodies (mAb) have specific targeting and robust anti-cancer properties. EGFR receptors overexpressed on the surface of CRC cells can be exploited to prepare active targeted NPs. Furthermore, cetuximab can bind specifically to EGFR-related structural domains expressed on the surface of multiple cancer cells. Drug couples formed by 5-Fu and cetuximab were loaded on the surface of Au NPs and exhibited enhanced tumor suppression and low toxicity (Fig. 8) [170]. EGFR-targeting panitumumab-modified PEG NPs acting on the HCT 116 mouse model deliver oxaliplatin specifically to the tumor site [171]. Some antibodies directed towards other membrane proteins are also employed to prepare targeted NPs. The up-regulated CD133 is expressed as a stem cell marker on the surface of several solid tumor cells. Mohd-Zahid et al. [172] used anti-CD133 mAb for targeted delivery of 5-Fu to CRC cells. The mAb targeting carcinoembryonic antigen (CEA) of CRC epithelial cells functionalizes the surface of PLGA-PEG NPs, thus effectively delivering paclitaxel to tumor tissue for killing tumor cells [173].

### Others

**Fig. 9:**
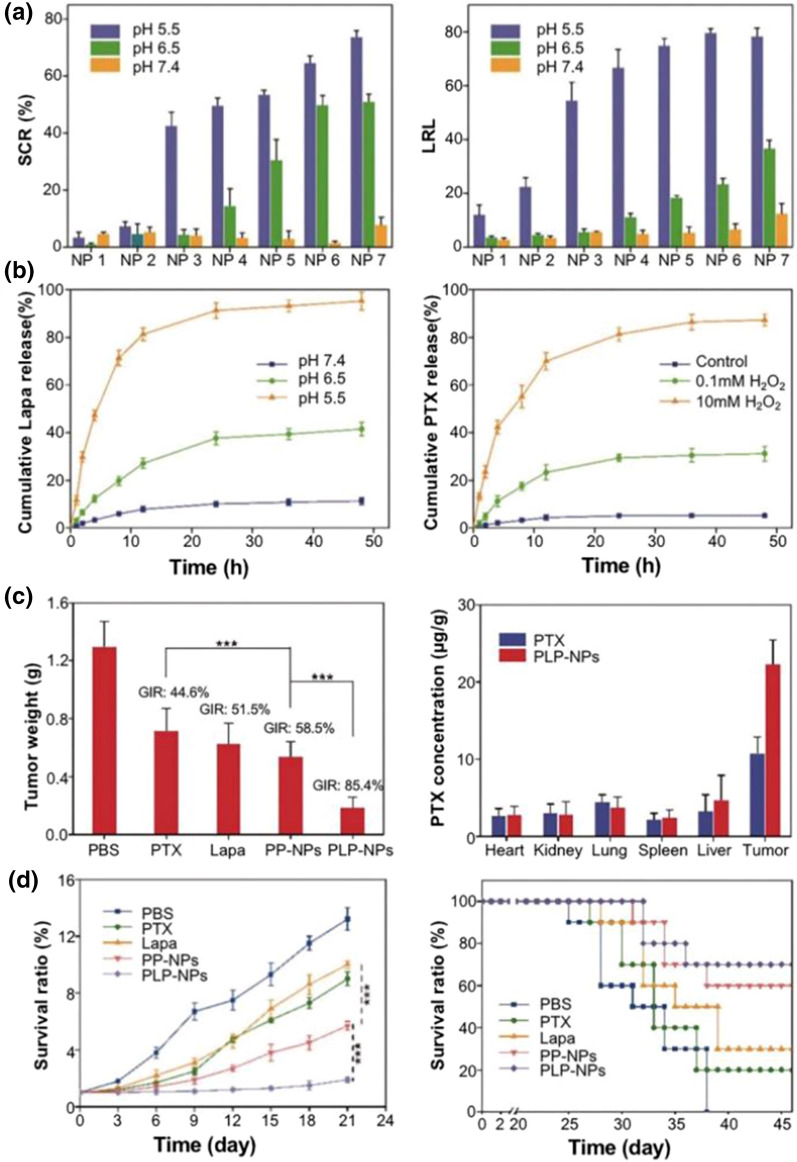
The composition of PLP-NPs with pH responsive PHis, ROS responsive thioketal and paclitaxel prodrugs can be targeted to treat multi-drug resistant CRC. **a** Size change ratio (SCR) and Lapa release level (LRL) of PLP-NPs after 8 h incubation at different pH conditions; **b** the curve of Lapa released from PLP-NPs at different pH conditions and that of PTX released from PLP-NPs at different concentrations of H_2_O_2_; **c** tumor weights and PTX levels in major tissues of HCT-8 tumor-bearing mice after 21 days of treatment in different groups. **d** Relative tumor volumes and survival rates of mice in different treatment groups (Reproduced with permission from [174]. © 2020 by *Na Chang et al.)*

Dual responsive NPs accumulate in tumor tissue through long in vivo circulation times and EPR effects, showing sensitive pH responsiveness in acidic TME or lysosomes and redox responsiveness upon internalization. Chang et al. [174] prepared pH/ROS dual-responsive NPs using pH-sensitive poly(l-histidine) (PHis) and β-lapaxone (Lapa) in combination with ROS-sensitive thioketal (Fig. 9). The experiment demonstrated that PHis protonation in the acidic lysosomal environment of dual-responsive NPs promoted the release of Lapa. Lapa promotes the further release of paclitaxel to kill cancer cells by generating large amounts of ROS, while the depletion of large ATP inhibits glycoprotein-mediated MDR. Dual responsive NPs can better target tumor cells for effect, representing a powerful weapon against MDR tumors.

The strategy of combining passive targeted NPs with active targeted NPs allows better delivery of drug-loaded NPs to tumor tissue. The pH-sensitive PEG lipid derivatives target tumor tissue with enhanced permeation and EPR effects, releasing NPs modified with targeting peptides to tumor cells, which can boost drug concentrations at tumor sites more effectively. PEG-lipid derivatives possess pH-sensitive imine bonds that undergo hydrolysis in the acidic pH of the tumor environment. Penetrating peptides targeting neovascularization and mitochondria can deliver miR-200 and irinotecan to tumor cells and demonstrate positive therapeutic effects of tumor growth inhibition and reduced systemic toxicity in a mouse model of in situ intestinal cancer [175]. Active targeting modifications can be also combined with photosensitizers to promote drug accumulation in tumor cells. The photosensitizer Ce6 was covalently linked through the reduction-sensitive bond maleimide thioether to address the problem of drug leakage during application. Surface modification of the tumor homing peptide tLyp results in more precise targeting to tumor tissue [176].

The ability to better assess the distribution of targeted drugs in normal and tumor tissues in vivo through traceable NPs has attracted the attention of DDS researchers. Superparamagnetic iron oxide NPs (SPIONs) have been applied in research. The practical applications of SPIONs are as follows: magnetic cell labeling, separation, and tracking; magnetic hyperthermia therapy and drug delivery; MRI of contrast agents for diagnostics. SPION can improve the contrast of normal and tumor tissues and show organ function or blood flow during in vivo diagnostics [177, 178]. The surface functionalization modification of SPION can improve stability. As a result, when loading chemotherapeutic drugs, drug aggregation in tumor tissue and normal tissue can be visualized by MRI [144]. Specific recognition mucin 1 (MUC1) is overexpressed on the surface of most tumor cells. The use of MUC1 as an active targeting ligand to deliver DOX with SPIONs and iron carrier couples provides a suitable DDS for tumor diagnosis and targeted anti-cancer drug delivery. Drug release assays demonstrated accelerated DOX release in an acidic environment, suggesting that a pH-responsive drug carrier could promote DOX accumulation at tumor sites while reducing non-specific cardiotoxicity [179].

In addition to passive targeted tumor tissues influenced by the acidic, reducing environment of the TME, external physical stimuli can also induce targeted drug release from NPs. Photodynamic therapy (PDT) involves the application of photosensitizing drugs and laser activation to treat tumors [180]. The irradiation of specific wavelengths to the lesion site can activate the photosensitizing drugs that selectively gather in the lesion tissue, triggering a photochemical reaction to destroy the lesion. Ce6 is a promising photosensitizer that can generate ROS when activated by specific wavelengths of light, resulting in potent induction of tumor cell death [181]. Chu et al. designed and prepared multifunctional nanophotosensitizers to overcome the hydrophobicity of Ce6 by wrapping Ce6 with pH-responsive micelles and modifying the EGFR-targeting peptide GE11 on its surface. It was applied to HCT-116 and SW620 xenograft tumor mouse models, and a 670 nm NIR laser was applied to irradiate the tumor sites, which exhibited significant anti-tumor effects [182]. It is also an example of a combination of active targeted and passive targeted strategies. Ultrasound can also be performed to assist in the targeted release of drugs into tumor tissue. The mechanical and thermal effects induced by ultrasound can destabilize the drug carrier structure and thus release the drug. Ultrasound can also penetrate the skin and most of tumor tissues, facilitating drug uptake by tumor cells [183]. Yan et al. developed an efficient siRNA delivery system that utilized chitosan derivatives loaded with anti-ß-catenin siRNA which can facilitate the release and action of siRNA from NPs through ultrasound [184].

Oral formulations of chemotherapy drugs improve patient compliance and avoid some of the systemic toxicity or phlebitis associated with injectable administration [185]. The gastrointestinal environment is complex, and drug absorption is influenced by pH, hydrolytic enzymes, food, and intestinal microorganisms [186]. The critical aspects of orally targeted NPs are avoiding gastrointestinal damage, increasing the retention time in the gastrointestinal tract, and reaching the colonic tumor area where they are efficiently and rapidly taken up by tumor cells. Therefore, NPs can accumulate in the tumor tissue and ultimately achieve efficient treatment [187]. Wang et al. [188] applied Eudragit S100 to wrap PLGA-NPs which were loaded with 5-Fu and the developed composite was able to reduce drug hydrolysis in the gastrointestinal tract and enhance drug loading at the tumor site. Li et al. [189] prepared polydopamine-coated nanodiamond (PND) utilizing the excellent drug-carrying ability of nanodiamond (ND) and the favorable biocompatibility and photothermal effect of polydopamine (PDA). Moreover, they loaded FA on the surface of PND to confer active targeting ability. The chitosan coating layer was also employed to maintain the stability of the drug carrier in the gastrointestinal tract. Inulin could protect the drug from the acidic environment of the stomach and upper gastrointestinal tract. It is degraded by inulinase in the colon for the controlled release of the drug employed as a drug carrier to target the colon [190].

RNA interference (RNAi) is a reaction of efficient and specific degradation of mRNA induced by double-stranded RNA, which can lead to sequence-specific gene silencing [191]. The discovery of RNAi mechanism provides a novel approach to the study of gene function in basic medical research. RNAi can target tumor-associated fusion gene transcription products, overexpressed oncogenes or apoptosis suppressors, tumor drug resistance genes, and tumor angiogenic factors and receptors for therapeutic purposes [192]. RNAi technology which targets tumor drug resistance genes can effectively overcome chemotherapy resistance due to gene mutations or modifications. BCL-2 can significantly inhibit tumor apoptosis and is overexpressed in a variety of tumor cells, and silencing BCL-2 expression by siRNA may lead to improved sensitivity of cancer cells to anticancer drugs [193]. Ray et al. prepared a PEI-based multi-drug co-delivery system that encapsulated doxorubicin, aspirin and BCL-2 siRNAs within NPs, and applied them to HCT116 cells to achieve superior anti-tumor effects [194]. Anti-EGFR monoclonal antibodies (mAB), such as cetuximab, are remarkably effective in CRC patients, while KRAS mutations always lead to the resistance to these drugs. The combination of anti-EGFR-mAB and siRNA targeting KRAS effectively suppressed KRAS protein expression in CRC cell lines, and reduced tumor volume and tumor weight effectively in a mouse implantation tumor model with cetuximab resistance mutation [195]. The preparation of appropriate targeted NPs can help siRNAs maintain stability in blood circulation, making RNAi technology the daybreak of overcoming tumor drug resistance.

In order to reduce tissue damage from chemotherapeutic drugs, delivery in the form of prodrugs is a promising solution. Yang et al. [196] conjugated cathepsin B-cleavable peptide (*Phe-Arg-Arg-Gly*, FRRG) with doxorubicin to form FRRG-DOX NPs and used the compound Pluronic F68 to further stabilize the prodrug delivery of NPs. CRC cells overexpressing histone protease B could activate the prodrug-loaded NPs and exert their anti-tumor effects, and CAP-NPs effectively inhibited tumor progression and attenuated toxic and inflammatory responses to normal organs through high cancer specificity in vivo.

## Conclusion and perspectives

Chemotherapy is one of the most prominent treatments for CRC. However, the complex composition of TME in CRC and the interaction between cellular and mesenchymal components constitute a tumor tissue with high cell density, dense extracellular matrix, and high osmotic pressure, inevitably preventing the entry of chemotherapeutic agents into the tumor cells for action [9]. Facile NPs passively target tumor tissue through the high permeability of imperfect vascular endothelium. Targeted NPs can modify the physicochemical properties of drugs, facilitate drug molecules to cross the physiological and pathological tissue barriers, and increase the local concentration of nanomedicines in the lesion. It improves drug efficacy while reducing side effects, thus achieving safer and more effective disease diagnosis and treatment and improving bioavailability [197]. The intelligent response of NPs is activated by the unique pH and redox state of TME to release the drug. The NPs remain stable in the neutral environment of normal tissues and reduce the damage to normal tissues [198]. Loaded tracers are also available to visualize tumor treatment effects [177]. Modification of NPs with specific ligands, aptamers or antibodies can enable them to bind to antigens or receptors particular to the surface of tumor cells, which is the strategy of active targeting. It allows the specific identification of tumor cells, improving the precision of tumor treatment and reducing the unnecessary side effects on normal tissues and organs.

Currently, the majority of studies on targeted NPs are still in the animal testing stage. Targeted NPs for CRC treatment still have a long way to go from research to clinical application. Researchers need to take into account the changes in vascular-based permeability and the potential damage to normal tissue from long circulation times when developing facile NPs that utilize EPR effects. The preparation of active targeted NPs has to address how to reduce off-target effects and perform large-scale reproducible preparation and screening of nanomedicines. The design of vectors for targeted NPs requires favorable biocompatibility and degradability, and excellent stability of physical and chemical properties. And the carrier degradation time should correspond to the frequency of administration to avoid adverse effects due to polymer accumulation. Targeted NPs are easy to store and maintain stable physicochemical properties over time. Most importantly, the tumor tissue needs to be accurately targeted, thus achieving superior anti-tumor effects without damaging normal tissue. All these issues require consideration in the process of targeted NPs research. Until the emergence of new disruptive drug delivery technologies, the delivery of targeted NPs remains a top priority in the research of CRC chemotherapy DDSs. Nanomedicines promise to be a dramatic breakthrough in targeted delivery.

## Data Availability

Not applicable.

## Abbreviations

CRC: Colorectal cancer
5-Fu: 5-Fluorouracil
TME: Tumor microenvironment
CAF: Cancer associated fibroblast
ECM: Extracellular matrix
PG: Proteoglycan
GAG: Glycosaminoglycan
FN: Fibronectin
NP: Nanoparticle
DDS: Drug delivery system
EPR: Effects of permeation retention
TAM: Tumor-associated macrophage
CXCL12: CXC subfamily ligand 12
IL-6: Interleukin-6
VEGF: Vascular endothelial growth factor
αSMA: α-Smooth muscle actin
PDGFRα: Platelet-derived growth factor receptor α
MMPs: Matrix metalloproteinases
HA: Hyaluronic acid
Tregs: Regulatory T cells
GARP: Glycoprotein-A repetitions predominant
PDCD4: Programmed cell death factor 4
MDSC: Myeloid-derived suppressor cell
GM-CSF: Granulocyte-macrophage colony-stimulating factor
Th2: Type II helper T
LOX: Lysine hydroxylase
LN: Lysine hydroxylase
LYVE-1: Lymphatic endothelial receptor
ROS: Reactive oxygen species
HIF: Hypoxia-inducible factor
Ang-2: Angiopoietin-2
iNOS2: Inducible nitric oxide synthase 2
ET-1: Endothelin-1
NSCLC: Non-small cell lung cancer
HCC: Hepatocellular carcinoma
PEG: Polyethylene glycol
PLGA: Polylactide-ethyl lactide
PLA: Polylactic acid
PCL: Polycaprolactone
FA: Folic acid
PEDF: Pigment epithelium-derived factor
Adr: Adriamycin
PAA: Polyacrylic acid
DOX: Doxorubicin
SPIO: Superparamagnetic iron oxide
GSH: Glutathione
LA: Lipoic acid
Xyl: Xylan
Nic: Niclosamide
TfR: Transferrin receptor
FR: Folate receptors
dsRNA: Double-stranded RNA
oHA: HA oligosaccharides
EpCAM: Epithelial cell adhesion molecule
mAb: Monoclonal antibody
CEA: Carcinoembryonic antigen
MUC1: Mucin 1
Lapa: β-Lapaxone
PND: Polydopamine-coated nanodiamond
PDA: Polydopamine
RBCm: Red blood cell membrane
